# Validation of the posttraumatic stress disorder checklist – 5 (PCL-5) in a primary care population with high HIV prevalence in Zimbabwe

**DOI:** 10.1186/s12888-018-1688-9

**Published:** 2018-04-23

**Authors:** Ruth Verhey, Dixon Chibanda, Lorna Gibson, Jonathan Brakarsh, Soraya Seedat

**Affiliations:** 1 Zimbabwe Aids Prevention Project, 92 Prince Edward Road, Milton Park, Harare, Zimbabwe; 2 Zimbabwe Aids Prevention Project, Harare, Zimbabwe; 3 London School of Tropical Medicine and Hygiene, London, UK; 4 Say and Play Therapy Centre, Harare, Zimbabwe; 5 College of Health Sciences, Stellenbosch University, Stellenbosch, South Africa

**Keywords:** Validation, Posttraumatic stress disorder Checklist-5, PTSD, Trauma, HIV, Low and middle income countries

## Abstract

**Background:**

There is a dearth of validated tools measuring posttraumatic stress disorder (PTSD) in low and middle-income countries in sub-Saharan Africa. We validated the Shona version of the PTSD Checklist for DSM-5 (PCL-5) in a primary health care clinic in Harare, Zimbabwe.

**Method:**

Adults aged 18 and above attending the clinic were enrolled over a two-week period in June 2016. After obtaining written consent, trained research assistants administered the tool to eligible participants. Study participants were then interviewed independently using the Clinician Administered PTSD Scale (CAPS-5) as the gold standard by one of five doctors with training in mental health.

**Result:**

A total of 204 participants were assessed. Of these, 91 (44.6%) were HIV positive, 100 (49%) were HIV negative, while 13 (6.4%) did not know their HIV status. PTSD was diagnosed in 40 (19.6%) participants using the gold standard procedure. Using the PCL-5 cut-off of ≥33, sensitivity and specificity were 74.5% (95%CI: 60.4–85.7); 70.6% (95%CI: 62.7–77.7), respectively. The area under the ROC curve was 0.78 (95%CI: 0.72–0.83). The Shona version of the PCL-5 demonstrated good internal consistency (Cronbach’s alpha = 0.92).

**Conclusion:**

The PCL-5 performed well in this population with a high prevalence of HIV. There is need to explore ways of integrating screening tools for PTSD in interventions delivered by lay health workers in low and middle-income countries (LMIC).

## Background

In sub-Saharan Africa, people living with HIV (PLWH) have a high rate of post-traumatic stress disorder (PTSD) [1, 2]. The negative experience of being HIV positive is cumulative with PLWH exposed to multiple HIV-related stressors and losses [3] which can lead to PTSD symptomatology.

Stressors, both acute and chronic, that people in LMIC are exposed to often occur on a daily basis. Political and economic instability, including poverty and disparity with its consequences of food scarcity and lack of access to medical care are some of the stressors. Furthermore, lack of education, interpersonal violence (IPV), and exposure to weather phenomena due to climate change can contribute to poor mental health outcomes including stress related disorders [4]*.*

Women are reported to be at greater risk of developing symptoms of PTSD [5] with cumulative effects of multiple traumas being common and associated with worse psychiatric and other chronic medical comorbidity [6–8].

In South Africa, PLWH have been found to have high prevalence of persisting psychiatric disorders with PTSD rates at follow-up of 20% being associated with a longer duration of infection and low baseline functionality [9]. The incidence of HIV-related PTSD in a cross-sectional study in the Western Cape, South Africa, was found to be at 40% [10]. Receiving an HIV-diagnosis was experienced as a traumatic index event for 36.4% in a population of recently diagnosed persons [9, 11].

The use of different PTSD assessment tools as well as the diversity with which traumatic events have been defined have contributed to varying outcomes in studies of PTSD [12]. Receiving a life-threatening diagnosis meets the threshold for consideration as a traumatic event for the development of PTSD [13] and was included in the Diagnostic and Statistical Manual of Mental Disorders DSM-IV (4th ed.; DSM-IV; American Psychiatric Association, 1994).

In general, too little is known about the prevalence of PTSD and PTSD related to HIV infection and its impact on health outcomes such as, health seeking behavior, treatment adherence and quality of life in sub-Saharan Africa. Furthermore, validated tools are scarce. For Zimbabwe, efforts have been made to validate screening tools for common mental disorders such as depression and anxiety for use in primary health care settings but none have been validated for PTSD [14] therefore this study seeks to address this deficit. The most appropriate current screening tool which based on DSM-5 is the Posttraumatic Stress Disorder Checklist (PCL-5) [15] which has not been validated in Africa. The Posttraumatic Stress Disorder Checklist (PCL) was developed at the National Center for PTSD [15]. It is a self-report measure that is widely used in western contexts and has been found to have very good psychometric properties in various settings [16–20].

## Methods

The validation exercise was carried out as a cross-sectional study at the largest clinic in the suburb of Mbare near the central business district of Harare, Zimbabwe.

Adults aged 18 and above attending the clinic were enrolled over a two-week period in June 2016. Pregnant women in their last trimester and women within the 3-month postnatal period were excluded to exempt postnatal depression [21], as were those who were unable to understand the purpose of the study.

After having obtained written consent, research assistants trained in the use of tablet computer facilitated data collection, administered the tool. Study participants were then clinically assessed by one of five doctors trained in mental health using the CAPS-5 (Clinician administered PTSD scale – 5) as the gold standard.

The study was approved by the medical research council of Zimbabwe (MRCZ, reference MRCZ/A/1732) and by the Health Research Ethics Committee at Stellenbosch University (reference S14/05/102).

### Sample size

We aimed to recruit a representative sample of *n* = 150 patients from a primary care clinic. We calculated that a minimum of 75 participants who scored positive on the reference standard for PTSD (CAPS-5) and 75 who scored negative would provide good precision for performance indicators for the PCL-5 achieving a sensitivity of 74.5% (95%CI:60.4–85.7%); specificity of 70.6% (95%CI:62.7–77.7%), positive predictive value (PPV) of 45.8% (95%CI:34.8–57.1%) and negative predictive value (NPV) of 89.3% (95%CI:82.3–94.2%).

### Translation of tools

The PCL-5, the LEC-5 (Life Events Checklist - 5) and the clinician administered PTSD scale for DSM-5 (CAPS-5) were translated from English into the local language Shona by a bilingual clinical social worker, and a bilingual psychiatrist (DC).

This draft Shona version was reviewed by a team of five lay health workers (LHW) working in a primary care mental health program called the Friendship Bench [22], five nurses working in the psychiatric ward of Harare Central Hospital together with a psychologist.

This phase focused on ensuring contextual equivalence to the original versions based on their understanding and use of local terms for trauma symptomatology.

An independent language expert back-translated the Shona version into English. The first author together with the social worker and the psychiatrist examined both original and back-translated versions and resolved any discrepancies by consensus. Translation and back-translation were carried out using a standard approach [23].

#### The PCL-5

The PCL was revised to match the adapted DSM-5 criteria for PTSD. The PCL-5 features an adapted answer scale ranging from 0 = not at all to 4 = extremely, thus making the theoretical lowest score 0. It measures 4 symptom clusters; the original clusters intrusion, avoidance and hyper-arousal and the added cluster of negative alterations in cognition and mood with three items (blame, negative emotions, and reckless or self-destructive behavior). Item scores can be summed for an overall severity score as well as for individual symptom cluster sums. A PTSD diagnosis can be made provisionally considering items rated 2 = moderately or higher as according to the DSM-5 diagnostic rule (at least one B, one C, two D, and two E symptoms present). In an empirical calibration, Blevins et al. (2015) found the psychometric properties for the PCL-5 for a US American college convenience sample with subjects who self-identified as having PTSD as follows: internal consistency α = 0.94 and test-retest reliability *r* = 0.82, 95% CI [0.71, 0.89] [16].

#### Clinician administered PTSD scale (CAPS-5)

The Clinician-administered PTSD scale CAPS-5 for DSM-5 [24] was used as the gold standard. It was derived from the CAPS for DSM-IV [25] and adjusted for the changes of the PTSD diagnosis in DSM-5 (American Psychiatric Association, 2013). The CAPS-5 is a structured clinical interview which allows the clinician to make a diagnosis of PTSD according to the criteria described in the DSM-5. Furthermore, overall symptom severity as well as global, social, occupational and personal impairment are assessed. The main Criterion (A), the traumatic event, is assessed with the added Life Events Checklist for the DSM-5 (LEC-5) [26].

#### The life events checklist for DSM-5 (LEC-5)

The LEC-5 is a self-report questionnaire asking for the prevalence of 16 potentially traumatic life-time events plus an added open category (“any other very stressful event or experience”) with five answer categories [26]. We used the LEC-5 in combination with the PCL-5.

#### SSQ-14

The Shona Symptom Questionnaire (SSQ-14) [27] was developed and recently re-validated in Zimbabwe in a HIV-population [14]. Most of the items are common to those found in tools for depression worldwide such as sleep disturbance and suicidal thoughts; others are local idioms of emotional distress including ‘thinking too much’. Participants are asked if they have experienced a list of common mental health symptoms in the past week. Each of the 14 items is scored dichotomously as yes (1) or no (0). With the optimal cut-off of ≥9, the sensitivity and specificity for the SSQ-14 against a diagnosis of depression and/or general anxiety were 84% (95% CI:78–89) and 73% (95%CI:63–81), respectively. Internal reliability was high (Cronbach α=0.74) [14].

### Training procedure

Study personnel (four research assistants, six LHWs and five medical doctors) working in the psychiatric unit of Harare Central hospital attended a two-week training using a guide initially developed by the authors (RV and DC). The research assistants were trained in data collection methods using the socio-demographic forms and the screening tools. The medical doctors were trained in the use of the CAPS-5 through a discussion forum led by RV which involved going through the diagnostic criteria, building consensus on how to manage clinically severe cases during the validation, and procedures for ensuring fidelity. RV and DC observed the doctors during role-play using the tool.

All raters were involved in a pilot study with patients visiting the psychiatric outpatient unit. Cohen’s Kappa was found to be high (k = 0.91) using a random sample size of *n* = 26. Participants in the pilot study were different from the participants of the validation exercise. The referral pathways for participants meeting criteria for PTSD and other acute medical conditions were determined as that they should be seen by the medical officer first, for assessment, before being referred to a tertiary psychiatric facility if needed.

### Data collection

Stage 1: Every morning during the study period, an appointed research assistant obtained the register of all adult patients waiting to be seen. The research assistant randomly selected clinic attendees based on a computer-generated random number sequence. Fifteen randomly selected participants were invited at one time to a quiet and private space where eligibility was determined. Informed written consent was sought from all those eligible. The four research assistants administered the SSQ and the PCL-5 to participants in randomly assigned alternating questionnaire order and also collected socio-demographic information such as age, gender, HIV status, marital and employment status. The interviews took 20–30 min and were conducted in a quiet space designated for the study team. Although the PCL-5 including the LEC and the SSQ are self-report tools, we chose to have them administered by trained research assistants as it was found in prior validation exercises using the same approach that tablet computer-use was not familiar to most of the clinic attendees [21].

Stage 2: Following administration of the screening tools, participants were referred to one of five medical doctors who conducted the CAPS-5. The doctors were blinded to the screening data. Each doctor asked the participant to recall the index event that was chosen, or the worst event if several were indicated, from the LEC-5 for the subsequent clinical interview. The CAPS-5 interview lasted 30–60 min in a private and quiet room. In case of a participant not reporting any event, the interview was not carried out.

All those needing acute psychiatric care were further assessed for treatment and referral to the psychiatric hospital.

### Data management

All data was double entered onto a password-protected database using Stata (version 13). No participant identifiable information was entered into the database. Ethical considerations and confidentiality for all participants were respected in accordance with Medical Research Council of Zimbabwe guidelines.

### Analysis

The performance of the PCL-5 was measured against the CAPS-5 as the gold standard. We estimated the sensitivity, specificity, positive predictive value (PPV) and negative predictive value (NPV) for different cut-points. The optimal cut-point was chosen to deliver a good balance between sensitivity and specificity. Results were presented in the form of a ROC curve which plots the true positive rate (sensitivity) against the false positive rate (specificity) [28]. The area under a ROC curve (AUC) quantifies the overall ability of the test to discriminate between those individuals with the outcome and those without the outcome.

Internal reliability was estimated using Cronbachs’ α. All analyses were conducted using Stata (version 13).

## Results

### Sample description

A total of the 204 (74.1%) participants were recruited during a 2-week period from a total of 275 who had been invited to take part. Seventy one were not illegible or declined participation. Of the final participants, 163 (85.3%) were female, 133 (69.6%) were married, and 139 (59%) had completed secondary education (Table 1). Overall sample mean age was 34 years. There were 108 (65.5%) participants who were unemployed. HIV sero-status was known for 191 (93.6%) participants, of whom 91 were HIV positive (44.6%). 13 (6.4%) did not know or refused to reveal their HIV status. Out of the 91 participants who were HIV positive, 79.1% were female. Being aged 40 and above (OR 15.03 CI6.35–35.57), being widowed (OR CI8.41 2.75–25.70) and having a SSQ 9 and above (OR 2.15 CI1.19–3.87) were associated with HIV positive status, respectively (Table 1).

**Table 1:** Socio-demographic characteristics of all participants by HIV status (*n* = 191)^b^

Characteristic	HIV positive (*n* = 91)		HIV negative (*n* = 100)		Logistic regression^a^		
	N	%	N	%	OR	95% CI	*p*-value
Gender							0.02
Male	19	20.9	9	9.0	1	–	
Female	72	79.1	91	91.0	0.37	(0.16–0.88)	
Age group							< 0.001
< 30	16	17.6	63	63.0	1	–	
30–39	33	36.3	26	26.0	5	(2.36–10.60)	
40+	42	46.2	11	11.0	15.03	(6.35–35.57)	
Marital status							< 0.001
Married	54	59.3	79	79.0	1	–	
Single	14	15.4	17	17.0	1.2	(0.55–2.65)	
Widowed	23	25.3	4	4.0	8.41	(2.75–25.70)	
Education							0.009
Less than ‘O’ level	45	49.5	31	31.0	1	–	
O′ level or more	43	50.6	69	69.0	0.46	(0.25–0.83)	
Current employment status							0.11
Unemployed	46	50.5	62	62.0	1	–	
Permanent FT or PT	8	8.8	3	3.0	3.59	(0.90–14.30)	
Casual/self-employed	37	40.7	35	35.0	1.42	(0.78–2.59)	
Main income source							< 0.001
Own business/salary	58	65.2	40	40.4	1	–	
Partner/family	22	24.7	51	51.5	0.3	(0.16–0.57)	
No income	9	10.1	8	8.1	0.78	(0.28–2.18)	
Suffer from chronic illness							< 0.001
No	10	11.0	66	66.0	1	–	
Yes	81	89.0	34	34.0	15.72	(7.23–34.18)	
Reason for clinic visit							< 0.001
HIV-related	37	40.7	1	1.0	1		
Routine/family/antenatal	34	37.4	54	54.0	0.02	(0.00–0.13)	
Other reason	20	22.0	45	45.0	0.01	(0.00–0.09)	
Negative life events in last 6 months							0.21
No	20	22.0	30	30.0	1	–	
Yes	71	78.0	70	70.0	1.52	(0.79–2.93)	
SSQ ≥ 9							0.01
No	46	50.6	68	68.7	1		
Yes	45	49.5	31	31.3	2.15	(1.19–3.87)	
PCL ≥ 33							0.09
No	49	53.8	66	66.0	1		
Yes	42	46.2	34	34.0	1.66	(0.93–2.98)	

^a^univariate logistic regression for outcome HIV+

^b^Participants who did not know or reveal their HIV status excluded (*n* = 13)

Forty-two (46.2%) of the HIV positive participants (*n* = 91) scored equal or above the cut-off score for PTSD. On the measure of depression (SSQ-14), 45 (49.5%) of this group scored above cut-off.

Amongst all 191 participants for whom the HIV status was known, 76 (39.7%) scored equal or above cut-off for PTSD as measured by the PCL-5.

### Traumatic events reported using LEC-5

On the self-experienced answer category, participants (*n* = 204) were asked to indicate all the events that happened to them in their entire life. The following five index events were reported as most common: physical assault [reported by 132 participants (64.7%)], the open category any other very stressful event or experience [130 participants (63.7%)], sudden, unexpected death of someone who was close to the participant (113 participants, 55.4%), life-threatening illness or injury (89 participants, 43.6%), as well as severe human suffering (86 participants, 42.2%).

### Traumatic events reported in the CAPS-5

In the clinical interview participants reported a similar distribution of events as the LEC-5. A total of 40 (19.6%) cases of PTSD were identified using our gold standard procedure. Qualifying traumatic events reported were categorized as follows: victim of physical, often combined with sexual assault (18 cases, 45%), being diagnosed with HIV as a life-threatening illness in 13 cases (32.5%) and having experienced sudden death of a person close to the participant in 9 cases (22.5%).

### Performance of the PCL-5 against the CAPS-5

A cut-off of ≥33 provided the highest proportion of participants correctly diagnosed compared with the CAPS-5 instrument. With this cut-off, sensitivity was 74.5% (95% CI: 60.4–85.7) and specificity was 70.6% (95% CI: 62.7–77.7) (Table 2).

**Table 2:** Sensitivity, specificity, PPV & NPV for PCL-5 cut-off ≥33 for whole sample and for PLWH only

	Sensitivity (95%CI)	Specificity (95%CI)	PPV (95%CI)	NPV (95%CI)
N = 204	74.5% (60.4–85.7)	70.6% (62.7–77.7)	45.8%(34.8–57.1)	89.3% (82.3–94.2)
N = 91 PLWH only	78.6% (59.0–91.7)	68.3% (55.3–79.4)	52.4% (36.4–68.0)	87.8% (75.2–95.4)

The PPV was 45.8% (95% CI: 34.8–57.1) and the NPV 89.3% (95% CI: 82.3–94.2) with Cronbach’s α = 0.92.

After stratification by HIV status the sensitivity for PLWH was 78.6% (59.0–91.7), and the specificity was 68.3% (55.3–79.4). The PPV was 52.4% (36.4–68.0), the NPV 87.8% (75.2–95.4) using the cut of ≥33.

**Fig. 1:**
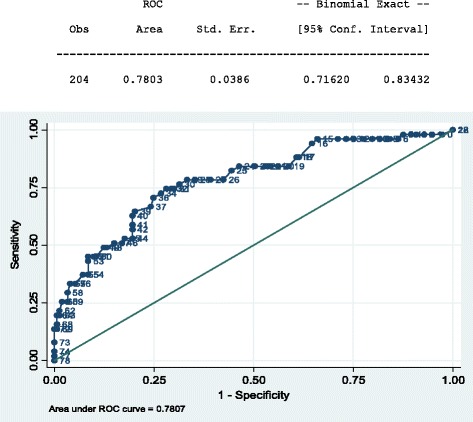
ROC curve for PCL-5 against CAPS 5 (*n* = 204)

The ROC curve for the performance of the PCL-5 gave an AUC of 0.78 (95% CI: 0.72–0.83) (Fig. 1).

## Discussion

The primary goal of this study was to validate the PCL-5 as the most current, DSM-5 keyed, PTSD measure (5th ed.; DSM-5; American Psychiatric Association, 2013) and to determine its utility and applicability in our context. We have validated other relevant tools for our work in CMD [14] but none for PTSD specifically.

The PCL-5 has not been validated within a comparable setting before, focusing on a population with high HIV prevalence.

The optimum cut-off score was found to be ≥33, a value which gave us a sensitivity of 74.5% (95%CI 60.4–85.7) and a specificity of 70.6% (95%CI 62.7–77.7). There was a difference after stratification according to HIV status [sensitivity = 78.6% (95%CI 59.0–91.7) and specificity = 68.3% (95%CI 55.3–79.4)]. This suggests the tool is more sensitive and less specific for PLWH compared to the results for the whole sample. This is further supported by the positive and negative predictive values which were 45.8% (34.0–57.1) and 89.3% (82.3–94.2) respectively for all participants (*n* = 204). The ROC curve (Fig. 1) gave an area under the curve (AUC) of 0.78 (95% CI0.716–0.834). The sensitivity of the tool being higher amongst PLWH was also the case in our earlier validation of tools for CMD [14]. There was good internal consistency (Cronbach’s α = 0.92).

Our results show that it is feasible to validate PTSD tools in a low resource setting using locally trained researchers. Although the PCL-5 is originally developed in a high-income setting where the context is different, we have shown that using cross-cultural methods as in earlier validation studies [21] is possible for PTSD tool validation. The inclusion of LHWs who are the delivering agent of the Friendship Bench intervention also makes the eventual use of the PCL-5 tool in their work at primary care level further possible. Including staff at all levels of the health system is important when developing interventions as this contributes to a stronger buy-in and increases the chances of the tool being used by all [29]. The results of this validation study will enable local researchers working on epidemiological and intervention studies with a focus on PTSD to work with a tool validated using an evidence based approach.

The PTSD prevalence of 46% amongst seropositive participants highlights the close link between HIV-status and PTSD prevalence, therefore it may be necessary to include screening for PTSD in the expanding HIV clinics in the country. Currently PLWH are screened using the SSQ-14 [27] which shows some overlap of symptomatology with PTSD (Table 1), however, there is need for PTSD specific screening in view of the high prevalence of PTSD. The prevalence of PTSD for all participants (*n* = 204) according to the gold standard measure CAPS-5 was 19.6%. Community studies have shown that trauma exposure is higher, often multiple, and associated with several chronic physical conditions [30].

A positive HIV sero-status is described as a traumatic event [31, 32]. In our study, one third (32.5%) of the PTSD cases identified by the gold standard clinical interview were found to have HIV infection as the qualifying traumatic event.

Being found out to be HIV positive is often met with interpersonal violence by partners or family members [33] with anticipatory fear often leading to non-testing or non-following up of treatment seeking behavior [34].

Whereas HIV can arguably be managed effectively in Western countries, the situation is different for the developing world. Poverty and unemployment, lack of access to medication and medical care, unavailability of adequate food, housing, risk of exposure to illnesses and to being re-infected due to lack of awareness and risky sexual practices as well as exposure to stigma and stigma-related interpersonal violence all comprise daily traumatic events for the HIV population in Zimbabwe.

The cumulative effect of these stressors is seen in the high prevalence of CMD of 49.5% amongst seropositive participants.

The Friendship Bench Program has had a successful, targeted approach to CMD [35], however we realize that PTSD identification could contribute towards adequately addressing the needs of primary care patients.

The use of psychological interventions to address common mental disorders (CMD) in PLWH in sub-Saharan Africa, their scaling-up, as well as their monitoring and evaluation underscores the importance of administering validated tools in rigorously conducted clinical trials [36, 37].

The PCL-5 has been developed in a western setting, as have most other psychological tools. Traumatic events according to the DSM-5 are defined as events that are linked to actual or threat of death, serious injury, or sexual violence experienced by self or close others, witnessed as well as heard of. Given the living situation for the majority of the population in LMIC, in our view the definition of a traumatic event has to be broadened. In LMIC, a wider range of circumstances hold a potential life threat for the population, as seen in the burden of disease studies [38]. Furthermore, people in LMIC are often exposed to multiple traumas [39] and have therefore a pronounced need for adequate, accessible and evidence-based care.

### Limitations

The gold standard interviews were carried out by medical doctors who all had a minimum of 2 years of work experience in psychiatry but no formal training in psychiatry. They were trained and supervised in the use of the tool by the first author together with the second author. The duration of training was less in comparison to other settings [16].

Study participants were urban dwellers visiting primary care clinics, therefore generalization beyond primary care settings is not possible. Furthermore, most of the study participants were female (85% females in our sample) which is common in this setting as shown in previous work [35, 40, 41], therefore, our finding may not be gender-sensitive.

PCL-5 and SSQ were administered in interview format despite them being designed for self-administration. Interviews were conducted by trained research assistants as study participants were not familiar with self-administration on tablet computers which were used for the data collection. A limitation of this approach is that participants might have displayed a social desirability bias [42] and therefore decreased the validity of the responses.

We did not investigate aspects of trauma attribution, resilience and vulnerability of individuals in this study nor did we discuss possible co-morbidities, all these are seen as associated with the development of PTSD [43, 44].

## Conclusion

It is possible to validate screening tools for PTSD in a low resourced setting with a high HIV prevalence.. We found the PCl-5 to have a good internal consistency with Cronbach’s α **=** 0.92, and with a cut-off score of 33 it showed good performance. Furthermore, our cut-off score is in line with what is suggested by the authors of the PCL-5 in its revised civilian version [16].

There is a need for integrating PTSD screening in primary care due to the high rate of PTSD within our study populations (45% for HIV positive and 34.3% for HIV negative participants, respectively).

## Abbreviations

AIDS: Acquired immune deficiency syndrome
ART: Anti-retroviral Therapy
CAPS-5: Clinician administered PTSD scale – 5
CI: Confidence interval
CMD: Common mental disorders
DMS-5: Diagnostic and Statistical Manual of Mental Disorders 5th edition
HIV: Human immunodeficiency virus
IPV: Interpersonal violence
LEC-5: Life Events Checklist – 5
LMIC: Low and middle income countries
MRCZ: Medical research council of Zimbabwe
NPV: Negative predictive value
PCL-5: Posttraumatic Stress Disorder Checklist – 5
PLWH: People living with HIV
PPV: Positive predictive value
PTSD: Posttraumatic Stress Disorder
ROC: Receiver operator curve
